# Paediatric Sweet's syndrome with pulmonary involvement triggered by severe inflammatory bowel disease and emergent total abdominal colectomy with literature review

**DOI:** 10.1002/ski2.326

**Published:** 2023-12-30

**Authors:** Fatmah Alzahrani, Hannah Tolson, Elizabeth Dupuy, Harper Price

**Affiliations:** ^1^ Division of Dermatology Phoenix Children's Hospital Phoenix Arizona USA; ^2^ Mayo Clinic College of Medicine and Science Phoenix Arizona USA; ^3^ Department of Child Health University of Arizona College of Medicine Phoenix Arizona USA

## Abstract

Sweet's syndrome (SS) is a neutrophilic dermatosis characterised by the acute onset of erythematous papules or plaques and a constellation of symptoms including fever, leucocytosis, and histopathology demonstrating nodular, pustular, or diffuse infiltrate of neutrophils with marked papillary oedema. SS can be a manifestation of inflammatory bowel disease and often coincides with periods of disease flares. Only a few cases of SS associated with ulcerative colitis are reported in the literature, and no cases have involved pulmonary manifestations in the paediatric population. We present a case of a 14‐year‐old girl presenting with new onset ulcerative colitis requiring emergent colectomy with subsequent development of pulmonary SS. Treatment consisted of intravenous and oral corticosteroids and dapsone. SS should be considered in the differential diagnosis of prolonged fever with cutaneous involvement and systemic symptoms. Special consideration should be given to paediatric patients with extracutaneous manifestations, particularly pulmonary involvement.

## 1 INTRODUCTION

In 1964, Dr. Douglas Sweet coined the term ‘acute febrile neutrophilic dermatosis’ to describe the abrupt onset fever, leucocytosis, painful plaques, and dense neutrophilic infiltrates on histopathology.^1^ Known as Sweet's syndrome (SS), it can be seen with malignancy, infection, and inflammatory diseases. Common systemic symptoms include myalgias, arthralgias, and fever.^2^ Pulmonary involvement is a rare and severe manifestation; the development of interstitial infiltrates is often preceded by the cutaneous manifestations.^3^ SS is rare in the paediatric population, and pulmonary manifestations in the paediatric population are even more sporadic.^4^ We present the striking case of a 14‐year‐old girl with SS with pulmonary involvement in the context of new‐onset ulcerative colitis requiring emergent surgical resection.

## 2 CASE REPORT

**FIGURE 1:**
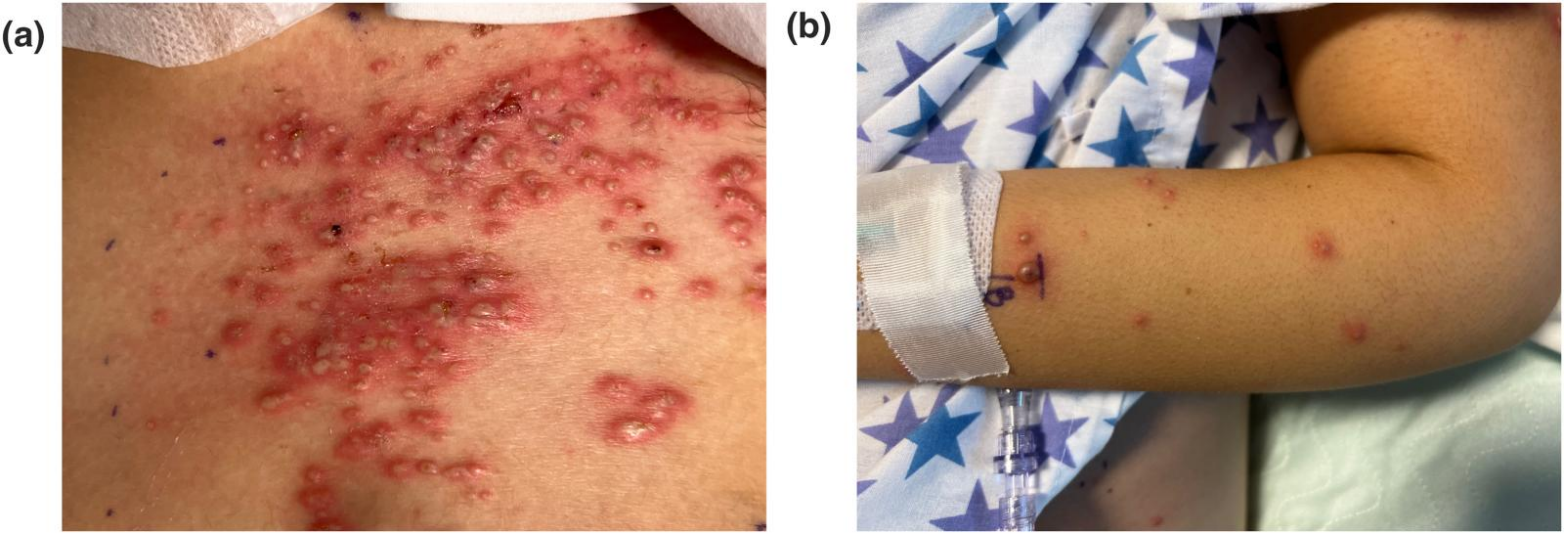
(a) Disseminated pustules on an erythematous base on abdomen; (b) recent pustule marked in ink from recent venipuncture.

A 14‐year‐old girl sought emergency treatment after 4 weeks of frequent bloody stools, severe abdominal pain, anaemia, fever, and tachycardia; she was diagnosed with ulcerative colitis. Oral corticosteroids, adalimumab, and infliximab were trialed with no response. Total parenteral nutrition and transfusion‐dependent, she underwent total abdominal colectomy with end ileostomy. On postoperative day 1, she developed leucocytosis, painful pustules on her entire abdomen (Figure 1a), and haemorrhagic papules at sites of line placement and blood draws (Figure 1b). Antivirals and antibiotics were commenced while awaiting the results of the infectious workup, as postoperative infection or abscess was the primary working diagnosis. Due to daily fevers and worsening labs, dermatology was consulted, and skin biopsies were obtained.

**FIGURE 2:**
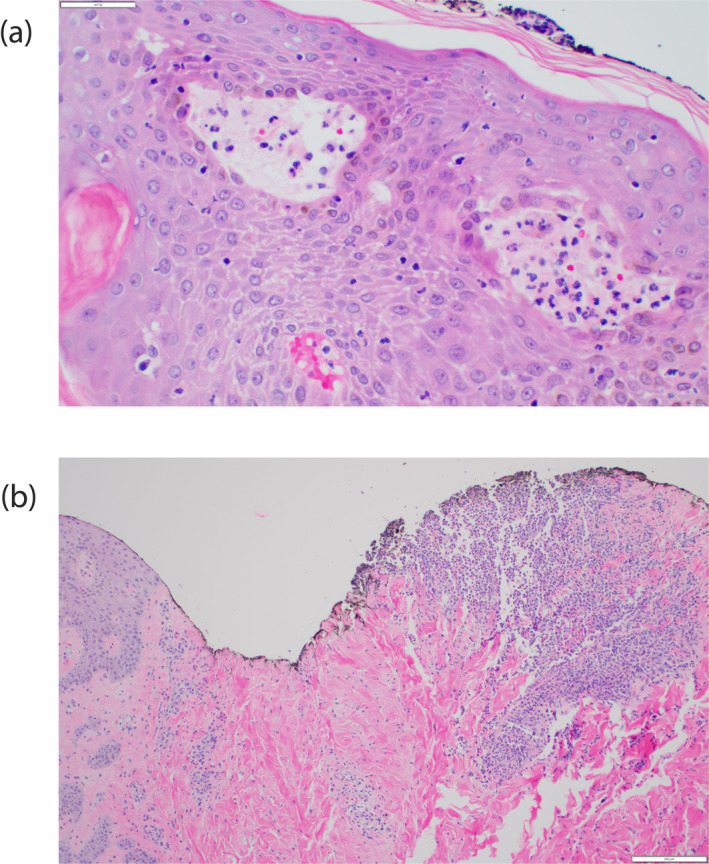
(a) Haematoxylin and eosin of skin biopsy showing papillary dermal microabscesses composed of neutrophils with leukocytoclastic debris; (b) haematoxylin and eosin of skin biopsy showing suprabasal acantholysis with neutrophilic inflammation.

Histopathologic evaluation showed epidermal and papillary dermal microabscesses composed of neutrophils with leukocytoclastic debris (Figure 2a). Acantholysis was seen in the suprabasal layer with neutrophilic inflammation (Figure 2b). Infectious workups were negative, including serial blood cultures, viral studies, tissue cultures, and special stains on histopathology. Intravenous solumedrol was initiated at a dose of 1 mg/kg/day with rapid improvement. Diagnosis of the pustular variant of SS was made. She was discharged on a slow tapering course of prednisone.

**FIGURE 3:**
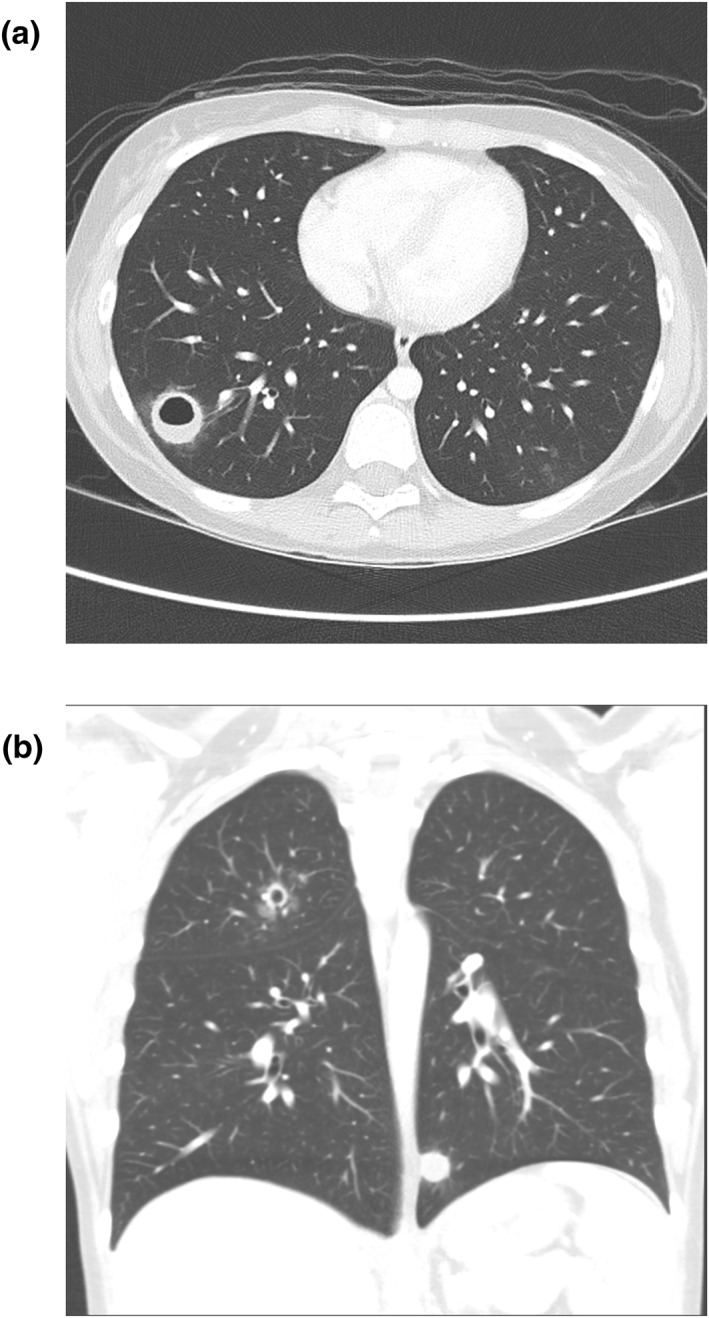
(a, b) Axial and coronal CT Chest with contrast showing right upper lobe cavitary lesion and left upper lobe nodule both with associated surrounding ground glass changes.

Fifteen days after discharge, during her steroid taper, our patient developed pleuritic chest pain, cough, fatigue, poor concentration, headache, and blood in her colostomy bag. Chest CT scan showed numerous bilateral pulmonary cavitary lesions with a peripheral soft tissue rim and ground glass opacities (Figure 3a,b). Pulmonary infectious workup was negative including negative tissue culture from lung biopsy and bronchial alveolar lavage. Tissue sampling of the lung showed neutrophilic abscesses and fibrinopurulent necrotic debris. She experienced a recurrence of painful pustules on her abdomen and legs which coincided with the development of pulmonary symptoms. Prednisone dosing was increased and dapsone was started. Two days later, her symptoms improved. Six months after discharge, she remains in remission on dapsone.

## 3 DISCUSSION

SS, or acute febrile neutrophilic dermatoses, is a hypersensitivity reaction typically seen in adults; children and adolescents comprise only 5%–8% of cases, and approximately 80 cases are reported in the literature.^4^, ^5^ SS diagnostic criteria require 2 major and at least 2 minor criteria (Table 1).^6^ The pathophysiology of SS is not well understood. An inciting event often precedes SS and prior literature has divided SS into categories based on aetiology: classic idiopathic, para‐inflammatory, paraneoplastic, pregnancy‐associated, and drug‐related.^7^ Up to 30% of paediatric SS cases are associated with chronic inflammatory conditions.^7^ A rare cause of paediatric SS is inflammatory bowel disease, and cutaneous symptoms typically correspond with periods of active disease.^8^ Disease associations by paediatric age group and recommended workups have been published.^9^

**TABLE 1:** Diagnostic criteria for the diagnosis of Sweet's syndrome.^6^

Major criteria	Minor criteria
1. Abrupt onset of typical cutaneous lesions 2. Histopathology consistent with Sweet syndrome	1. Preceded by one of the associated infections or vaccinations; accompanied by one of the associated malignancies or inflammatory disorders; associated with drug exposure or pregnancy
	2. Presence of fever and constitutional signs and symptoms
	3. Leucocytosis
	4. Excellent response to systemic corticosteroids

SS is not solely a skin disease and is well‐known to have extracutaneous manifestations. Arthralgias, conjunctivitis, and myalgias are common extracutaneous manifestations. Pulmonary manifestations are an unusual and severe complication of SS. Pulmonary involvement in SS manifests as neutrophilic alveolitis with corticosteroid‐responsive culture‐negative pulmonary infiltrates. Only about 5% of paediatric SS have lung findings on imaging.^10^ Five cases of pulmonary SS have been reported, and SS with pulmonary involvement triggered by inflammatory bowel disease has not been described to date. Although a recent sole case of a paediatric patient with pyoderma gangrenosum and pulmonary findings has been reported, there have been no cases thus far of inflammatory bowel disease‐triggered SS with pulmonary findings.^11^ (Table 2).

**TABLE 2:** Reported cases of Sweet's syndrome in the paediatric population, as well as 1 case of pyoderma gangrenosum.

References	Patient age	Sex	Past medical history	Pulmonary involvement	Infectious workup	Treatment
Collins et al.^12^	27 months	Male	None	Recurrent pneumonia since 5 months of age with worsening rash	Sputum culture positive for *H. Influenza* on bronchoscopy; repeat bronchoscopy at 27 months was unremarkable with sterile aspirate	Oral prednisone
Tzelepis et al.^9^	17 years	Male	Stage IV Hodgkin's lymphoma	Bilateral upper lobe pneumonia on chest CT	Blood and sputum cultures negative	IV methylpred for 2 days, PO prednisone
O’Regan et al.^13^	17 years	Male	Common variableimmunodeficiency	Acute pulmonary haemorrhage	Neutrophil count, LDH, Streptococcus pneumonia antigen negative	Monthly IVIG, PO dapsone
Arakaki et al.^5^	31 days	Female	None	Cough, respiratory distress, no focal infiltrates on CXR	Workup unspecified; patient treated with broad‐spectrum antibiotic and antiviral coverage	IV solumedrol for 3 days, PO prednisone for 10‐week taper
Hamilton et al.^3^	15 years	Female	Acute myelogenous leukaemia	Cough; ground‐glass opacities on chest CT in right lung base	Treated with IV cefepime and Amphotericin B empirically. Serologic testing for Histoplasma, Aspergillus, and Blastomyces and fungal polymerase chain reaction from both bronchoalveolar lavage (BAL) and skin biopsy negative	None
Martín‐Zamora et al.^11^	6 years	Female	None	Basal infiltrates with cavitation and pleural effusion in the right lung	Empirically treated with intravenous ceftazidime and clindamycin. Blood, sputum, BAL aspirate, and ulcer exudate cultures were negative for bacteria and fungi	IV methylpred, followed by slow taper
Our case	14 years	Female	Ulcerative colitis	Pleuritic chest pain, cough; pulmonary cavitary lesions and ground glass opacities	Serial blood cultures, viral studies, tissue cultures, and special stains on the histopathology were all negative	IV solumedrol, PO prednisone, and PO dapsone

Our patient met all major and minor criteria for SS in the perioperative period while in critical condition due to non‐treatment responsive ulcerative colitis. Although SS should be included in the differential in all patients who meet diagnostic criteria, infectious workup should be thorough and negative, as in our patient.

Recognition of pulmonary SS is paramount. Literature has shown that cases of SS with pulmonary involvement can cause bronchiolitis obliterans organising pneumonia, a rare, potentially fatal complication if left untreated.^9^ In conclusion, this case highlights SS as a rare multisystem disease in children, that may present with extracutaneous manifestations including pulmonary involvement.

## CONFLICT OF INTEREST STATEMENT

The authors declare no conflicts of interest.

## AUTHOR CONTRIBUTIONS

**Fatmah Alzahrani**: Investigation (lead); writing – original draft (lead). **Hannah Tolson**: Writing – original draft (equal); writing – review and editing (supporting). **Elizabeth Dupuy**: Writing – review and editing (equal). **Harper Price**: Writing – review and editing (equal).

## ETHICS STATEMENT

As the patient is under the age of 18, informed consent was obtained from the parents of the patient for publication of the case report.

## Data Availability

The data underlying this article cannot be shared publicly for the privacy of the individual about whom this case is.
